# Neuropsychiatric Involvement in Hospitalized COVID-19 Is Embedded in Multi-Organ Disease: A Retrospective Five-Axis Record-Derived Phenotype Atlas with Renal-Urinary Prognostic Dominance

**DOI:** 10.3390/pathogens15090979

**Published:** 2026-09-15

**Authors:** Claudia Daniela Lupu, Vlad-Dan Cotuțiu, Victoria Birlutiu

**Affiliations:** 1 Infectious Diseases Department, Academic Emergency Hospital Sibiu, 550245 Sibiu, Romania; victoria.birlutiu@ulbsibiu.ro; 2 Department of Parasitology and Parasitic Diseases, University of Agricultural Sciences and Veterinary Medicine of Cluj-Napoca, Calea Mănăștur 3–5, 400372 Cluj-Napoca, Romania; vlad.cotutiu@usamvcluj.ro; 3 AINIMAL SRL, Strada Stejarului 41D, 407280 Florești, Romania; 4 Faculty of Medicine, Lucian Blaga University of Sibiu, 550169 Sibiu, Romania

**Keywords:** COVID-19, multi-organ phenotype, neuropsychiatric manifestations, renal-urinary axis, electronic health records, ICD-10 phenotyping, clinical outcomes

## Abstract

Hospitalized COVID-19 frequently involves several organ systems at once, yet neuropsychiatric manifestations are often examined in isolation. In a retrospective single-center cohort study, a five-axis phenotype atlas was constructed (respiratory, cardiovascular, neuropsychiatric, hepato-gastrointestinal, and renal-urinary) from ICD-linked hospital records of 1361 adults hospitalized with confirmed SARS-CoV-2 infection at a Romanian county emergency hospital between March 2020 and January 2025. Candidate neuropsychiatric associations were evaluated under prespecified cross-axis adjustment, treatment- and indication-bias gates, penalized outcome models, and pandemic-era comparison. Multi-organ involvement was almost universal: 91.0% of admissions carried at least two axes. Neuropsychiatric positivity reached 22.9% but was dominant in 0.8%, while 98.1% of these patients carried another chronic organ axis. Of the 42 initial laboratory candidates, three correlated electrolyte associations remained after adjudication, suggesting that these associations were more consistent with shared renal-metabolic burden than with neuropsychiatric-specific biology. Renal-urinary involvement was independently associated with intensive care admission (OR 1.97) and in-hospital death (OR 2.58); neuropsychiatric positivity was not. The previously reported Late Omicron enrichment of severe neurological presentations remained evident after age and sex adjustment (adjusted OR 3.35) and was the only one of 35 prespecified claims retained as neuropsychiatric-specific. Renal-urinary rather than neuropsychiatric involvement carries the adjusted prognostic weight in hospitalized COVID-19, and, given the dependence of retrospective record-derived phenotypes on clinical documentation, multi-organ adjudication should precede organ-specific inference from hospital records.

## 1. Introduction

Coronavirus disease 2019 (COVID-19) is a multifactorial respiratory illness in which viral pneumonia, immune dysregulation, and multi-organ complications frequently co-occur [1]. During early pandemic waves, clinical priorities were dominated by acute respiratory failure, surging hospitalizations, and in-hospital mortality, limiting systematic capture of neurological and neuropsychiatric complications that emerge or worsen after discharge. As post-acute COVID-19 definitions have consolidated and acute mortality has fallen, persistent neurological, cognitive, psychiatric, and functional impairment is increasingly recognized as a major unresolved burden [2,3]. Electronic health record frameworks further expose persistence and coding gaps across health systems, motivating label-efficient phenotyping where structured ascertainment alone is incomplete [4,5]. Neurological and psychiatric complications were recognized early in the pandemic and remain clinically important in hospitalized adults. In the first Wuhan inpatient series, Mao et al. [6] reported neurological manifestations in 36.4% of patients, more often in severe disease. Subsequent cohorts confirmed that altered mental status, stroke, seizures, and psychiatric syndromes frequently accompany acute respiratory failure, coagulopathy, and multi-organ dysfunction rather than occurring as isolated brain-compartment events [7,8,9]. Pandemic-era acute-care studies likewise show that neurological spectra shift across variant periods, including rising delirium and encephalopathy in later waves despite lower overall severity [10,11]. These findings emphasize the need to distinguish temporal and systemic context from standalone neurobiological inference.

Although neuropsychiatric manifestations have been extensively described throughout the pandemic, an important question remains unresolved: whether the apparent neuropsychiatric signal reflects brain-specific pathology or the broader systemic response accompanying severe COVID-19. Distinguishing between these possibilities is essential for interpreting both laboratory and prognostic associations reported in hospitalized patients and provides the conceptual basis for the present study.

Inflammatory, coagulation, and neural-injury markers are often elevated in neurological presentations [12,13], and de novo central nervous system involvement in severe COVID-19 carries high in-hospital mortality alongside systemic risk markers [14]. Reviews of COVID-associated acute brain dysfunction similarly frame encephalopathy within sepsis-associated organ failure, endothelial injury, and blood–brain barrier disruption [8,9]. In large inpatient cohorts, impaired consciousness, delirium, and cerebrovascular events co-occur with elevations in routine inflammatory, coagulation, and renal markers [15,16]. Prospective neurological cohorts likewise link IL-6, LDH, ferritin, and troponin with in-hospital mortality among patients with documented neurological complications [12], while kidney biomarker panels track inflammation, severity, and mortality across admission trajectories [17]. Ward-level and intensive care unit data further show that changes in these same panels track acute delirium and coma [18]. In patients with severe COVID-19 pneumonia, serum neuron-specific enolase and electroencephalographic patterns stratify encephalopathy severity without establishing neuropsychiatric-specific biology [19]. This literature situates paraclinical signals within systemic disease severity. The complementary question, whether such associations remain neuropsychiatric-specific once concurrent organ involvement is explicitly modeled, requires a different analytic design and motivates the present study.

Reporting of neuropsychiatric COVID-19 phenotypes in Europe remains fragmented between multinational neurologist registries, national surveillance platforms, and single-center hospital series. The European Academy of Neurology ENERGY registry and linked multinational cohorts documented stroke, encephalopathy, and impaired consciousness among referred inpatients, with substantial short-term functional decline and in-hospital mortality [20,21]. Romanian hospital studies from Bucharest neurology-referral and northeastern infectious-disease settings have described heterogeneous acute neurological presentations and wave-dependent symptom patterns [22,23], while a central Romanian county-hospital study reported wave-stratified neuropsychiatric cluster prevalence from ancestral through Omicron-dominant periods [24]. Other neuro-COVID studies have examined biomarker- or imaging-enriched populations [25,26], highlighting the heterogeneity of ascertainment strategies and their limited comparability with record-derived phenotyping across all hospital admissions.

At the national level, Romania sustained one of the lowest COVID-19 vaccination coverages in the European Union, not exceeding roughly 40% by the end of 2021 [27]. It also carried among the highest pandemic mortality in the Union, with about 63,600 COVID-19 deaths by early 2022 and the third-highest cumulative excess mortality among member states by December 2021 [28].

The present study positions record-derived neuropsychiatric phenotypes within the multi-organ spectrum of hospitalized COVID-19. Using five prespecified organ axes, it evaluates whether neuropsychiatric paraclinical and prognostic associations remain interpretable as organ-specific after explicit adjustment for concurrent respiratory, cardiovascular, hepato-gastrointestinal, and renal-urinary involvement, treatment exposure, and documentation structure, and it compares the adjusted prognostic weight carried by each axis. This design separates findings specific to neuropsychiatric involvement from those shared with systemic disease through prespecified methodological adjudication rather than post-hoc filtering.

## 2. Materials and Methods

### 2.1. Design, Setting, and Aim

This retrospective observational cohort study included adults hospitalized with confirmed COVID-19 at Academic Emergency Hospital Sibiu, Romania, between March 2020 and January 2025. The aim was to determine where record-derived neuropsychiatric phenotypes sit within a broader multi-organ clinical and paraclinical phenotype atlas and whether they retain neuropsychiatric-specific paraclinical or prognostic associations once concurrent multi-organ involvement is explicitly modeled.

The prespecified primary organ axes were respiratory (RESP), cardiovascular (CARDIO), neuropsychiatric (NEUROPSY), hepato-gastrointestinal (HEPATO_GI), and renal-urinary (RENAL_URINARY). SYSTEMIC evidence was retained as a covariate layer rather than as a sixth primary phenotype family. All analyses were associative and observational. Causal, mediation, radiology-predictor, and spatial-biological claims were required to pass explicit downstream gates before entering final interpretation.

The analytic workflow had three sequential layers: (i) an initial symptom-cluster paraclinical discovery phase that generated the hypothesis set; (ii) construction of the five-axis organ atlas; and (iii) a prespecified claim-adjudication protocol.

### 2.2. Study Population, Data Sources and Linkage

The study population comprised *n* = 1361 hospitalized adults with confirmed SARS-CoV-2 infection and linked inpatient paraclinical records. Clinical variables included age, sex, pandemic wave, hospitalization duration, ICU admission, in-hospital mortality, oxygen-support level, and neuropsychiatric cluster assignments from the same hospital record framework described in Lupu et al. [24]. For the present analysis, this framework was extended with refined ICD acuity mapping, expanded phenotype evidence, and prespecified adjudication gates applied before final inference, using the same parent cohort described previously [24].

The source population comprised more than 5000 SARS-CoV-2-positive hospitalizations recorded at the study center between March 2020 and January 2025, from which the parent cohort of 1471 admissions was sampled as previously described [24]. Eligible admissions were adults with laboratory-confirmed infection, a hospital stay longer than one day, and a primary or secondary COVID-19 diagnosis (ICD-10 J12.8 or U07.1). Of the 1471 admissions, 1364 were successfully linked to the required paraclinical records. Of these, 1361 had complete information for the final organ-axis analyses and constituted the analytic cohort (Figure S1).

Paraclinical data came from the hospital laboratory information system and microbiology service. Extraction was performed using an updated version of the natural-language processing pipeline previously applied to this record system [24]. For each admission, laboratory parameters were summarized across all in-hospital measurements (mean, median, minimum, maximum, and measurement count) across hematology, biochemistry, coagulation, immunology, and microbiology domains. Microbiology was summarized as organism-detection flags and resistance profiles.

Patients were linked between clinical and paraclinical records using national patient identifier, primary ICD-10 diagnosis, and admission date. ICU admission and in-hospital death were analyzed as binary endpoints (192 ICU admissions, 14.1%; 128 deaths, 9.4%). Oxygen-support level was retained for sensitivity analyses.

### 2.3. Neuropsychiatric Subphenotypes and Initial Paraclinical Models

Neuropsychiatric outcomes were severe neurological (NE3), moderate neurological (NE2), mild psychiatric (PSY1), moderate psychiatric (PSY2), neuro-cardiovascular (NECA), combined neuropsychiatric (NEPSY3), aggregate neurological (NEURO), aggregate psychiatric (PSY), and neuropsychiatric organ-axis (NEUROPSY) positivity, using the previously published cluster definitions [24]. NE2 included headache, vertigo, dizziness, anosmia, ageusia, paresthesia, tremor, somnolence, dysarthria, and amnesia; NE3 included confusion, seizures, paresis, aphasia, hemiparesis, motor deficit, and obtundation; PSY1 comprised anxiety, insomnia, and depression; and PSY2 comprised psychomotor agitation and panic attacks. Syncope and hallucinations carried dual classifications and were assigned to the NECA and NEPSY3 clusters, respectively, with deduplication at the patient level [24]. The aggregate NEURO indicator was positive if NE2, NE3, NECA, or NEPSY3 was positive; the aggregate PSY indicator was positive if PSY1, PSY2, or NEPSY3 was positive. NECA and the aggregate indicators were retained for descriptive and era comparisons; subcluster outcome models were restricted to NE2, NE3, PSY1, PSY2, and NEPSY3. The NEUROPSY organ axis was assigned separately from ICD-linked acuity states and is distinct from these symptom-level indicators.

Initial paraclinical testing proceeded in two steps. First, each laboratory and microbiology variable was screened against each neuropsychiatric outcome using Mann–Whitney U or Fisher exact tests with Benjamini–Hochberg FDR correction within analyte category. Second, multivariate logistic models were fit with LASSO pre-screening (10-fold cross-validation), unpenalized logistic regression on selected predictors, variance inflation factor screening (threshold 5), and bootstrap stability assessment (1000 resamples for primary models). Continuous predictors were standardized; models were adjusted for age, sex, hospitalization duration, and pandemic wave, with ICU admission additionally forced for NE3, NEURO, and NEUROPSY models.

Parameters with fewer than 30% of patients measured were excluded from primary testing; 10–30% coverage was treated as exploratory. Primary reported models were NE3 standard and convergent-documentation variants, NE3 oxygen-support sensitivity, NE2, PSY1, NEURO, and NEUROPSY. Model coefficients, predictor lists, sample sizes, coverage exclusions, and sensitivity variants are in Table S1.

Pandemic era for later temporal analyses was defined as Early (waves 1–2), Mid/Delta (waves 3–4, spanning the Delta and early Omicron periods), and Late Omicron (wave 5, Omicron sublineages from July 2022 onward), with Early as reference (Table S2). These labels denote fixed calendar groupings of the previously defined waves [24] rather than variant attribution for individual admissions.

### 2.4. Multi-Organ Phenotype Assignment and Descriptives

Organ-axis positivity was assigned from ICD-linked acuity states (acute, chronic, acute-on-chronic) integrated with symptoms, consultation, medication, and chronic-context evidence frozen before paraclinical adjudication. Five binary axis flags (has_RESP, has_CARDIO, has_NEUROPSY, has_HEPATO_GI, has_RENAL_URINARY) were derived for all downstream models.

**Figure 1:**
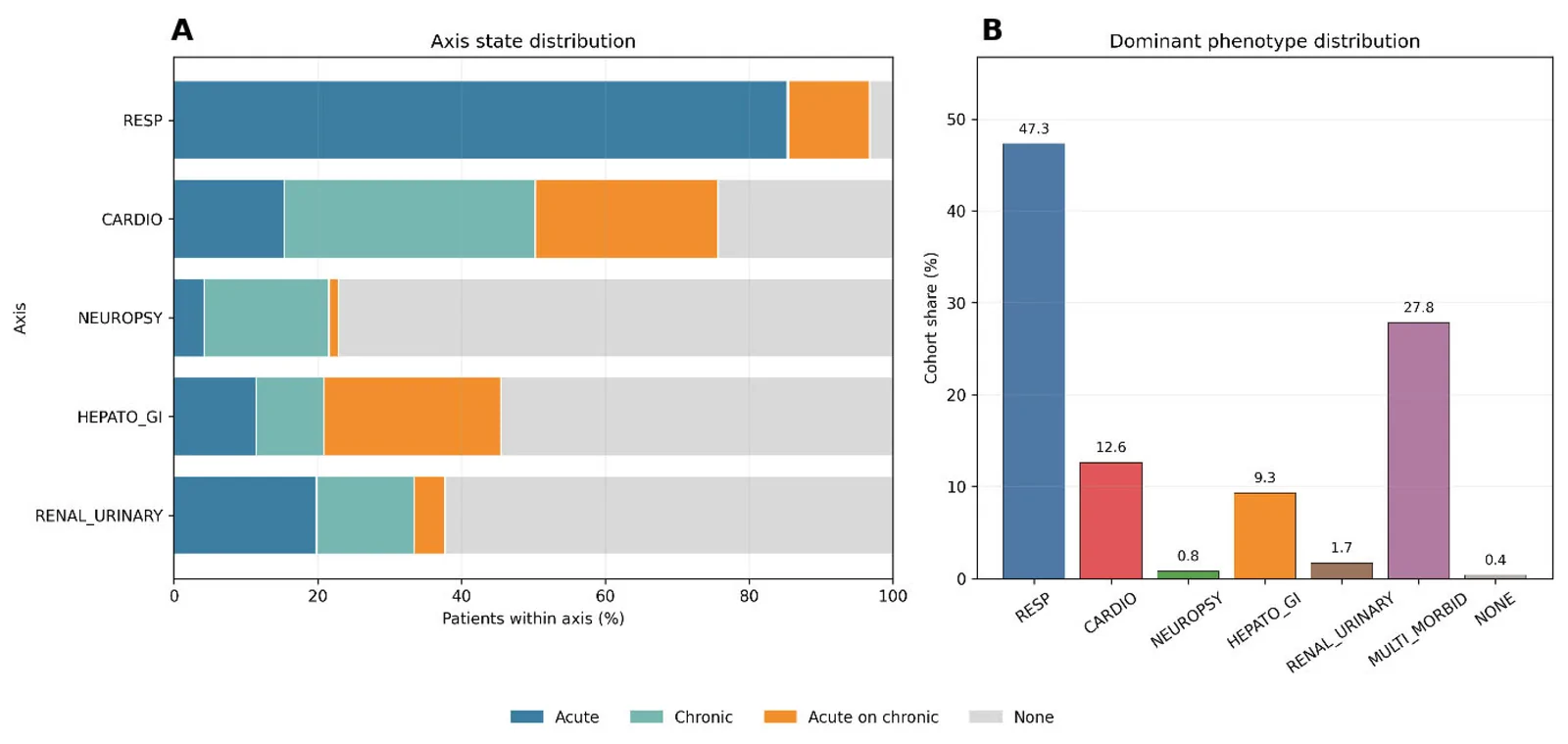
Multi-organ axis prevalence and dominant-phenotype distribution. (**A**) axis-positivity prevalence for the five organ axes. (**B**) share of admissions assigned to each dominant phenotype.

A mutually exclusive dominant-phenotype label (RESP, CARDIO, NEUROPSY, HEPATO_GI, RENAL_URINARY, MULTI_MORBID, or NONE) was assigned from ICD severity counts using a 3-2-1 reference score and a 30% dominance threshold, with alternative weighting schemes and thresholds examined in sensitivity analyses. Dominant-phenotype labels served descriptive subgroup assignment only (Table 1; Figure 1) and were kept separate from the binary axis-positivity flags used in outcome models; an admission could be renal-axis positive while its dominant phenotype was respiratory, cardiovascular, or multi-morbid.

Cohort descriptives and cross-axis overlap (Jaccard matrices for any-state, acute, chronic, and respiratory-excluded views) are in Table 1 and Tables S3 and S4.

### 2.5. Prespecified Adjudication Analyses

After initial paraclinical models, every candidate association was re-tested under the modules in Supplementary Table M1. Modules were applied in fixed order; a positive screen at one layer did not bypass later layers. The aim was to establish which initial neuropsychiatric claims still supported neuropsychiatric-specific interpretation once co-occurring organ disease, treatment exposure, and documentation structure were explicitly controlled (Table S5).

The geographic module tested access and documentation patterns, not biological mechanism. Since specialist neurological and psychiatric services are unevenly distributed across the county, differential pre-hospital documentation of chronic neuropsychiatric disease would produce a residential gradient in organ-axis positivity by distance, altitude, or urban versus rural residence.

### 2.6. Outcome Estimators and Separation Handling

**Figure 2:**
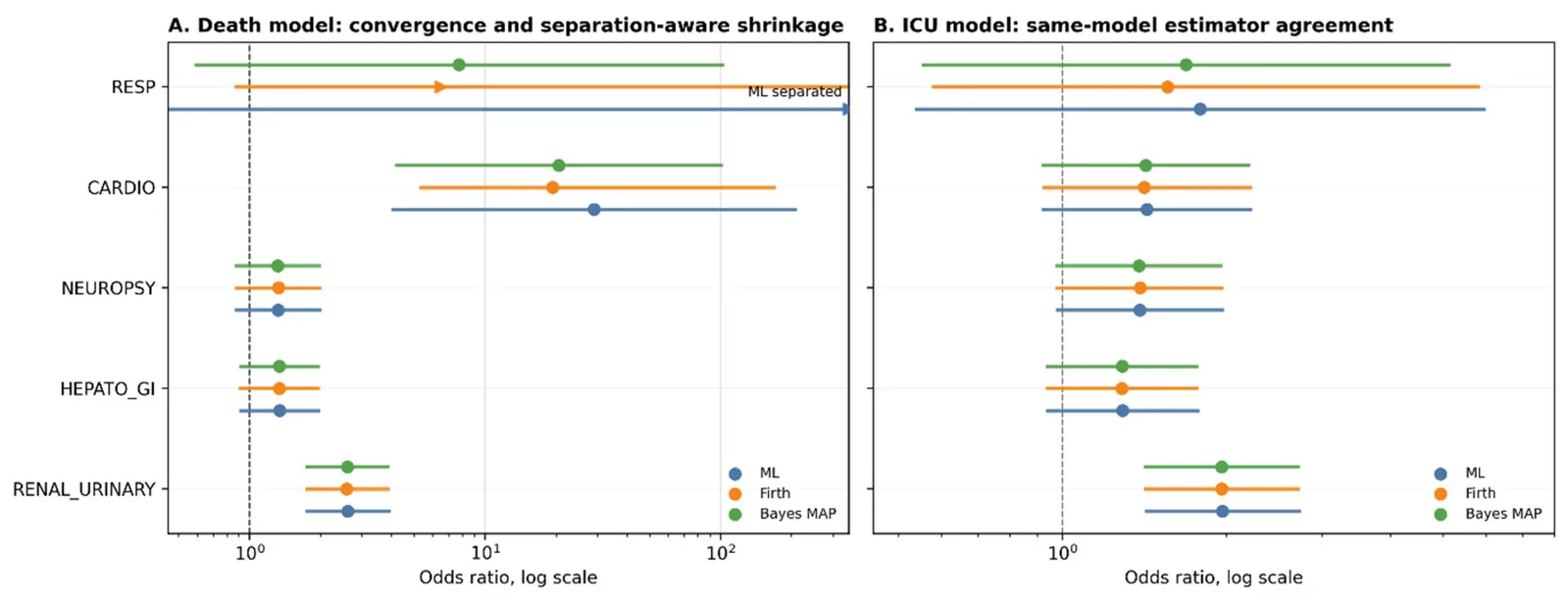
Comparison of maximum-likelihood, Firth-penalized, and Bayesian MAP estimates for mutually adjusted organ-axis associations with (**A**) in-hospital death and (**B**) ICU admission. Odds ratios are shown on a logarithmic scale; the maximum-likelihood respiratory death estimate was non-identifiable because of separation. The dashed vertical line marks an odds ratio of 1 (no association); arrowheads indicate confidence limits extending beyond the plotted range.

Death models with quasi-complete separation (notably near-universal respiratory axis positivity and near-ceiling cardiovascular prevalence among decedents) were re-estimated using Firth penalized logistic regression (primary death estimator) and weakly informative Bayesian logistic regression with Cauchy priors on rescaled slopes (maximum a posteriori fit with Laplace intervals). These estimators used the same covariates and admissions as the primary maximum-likelihood models. Agreement across estimators was interpreted as robustness of one model specification, not independent replication (Figure 2).

**Figure 3:**
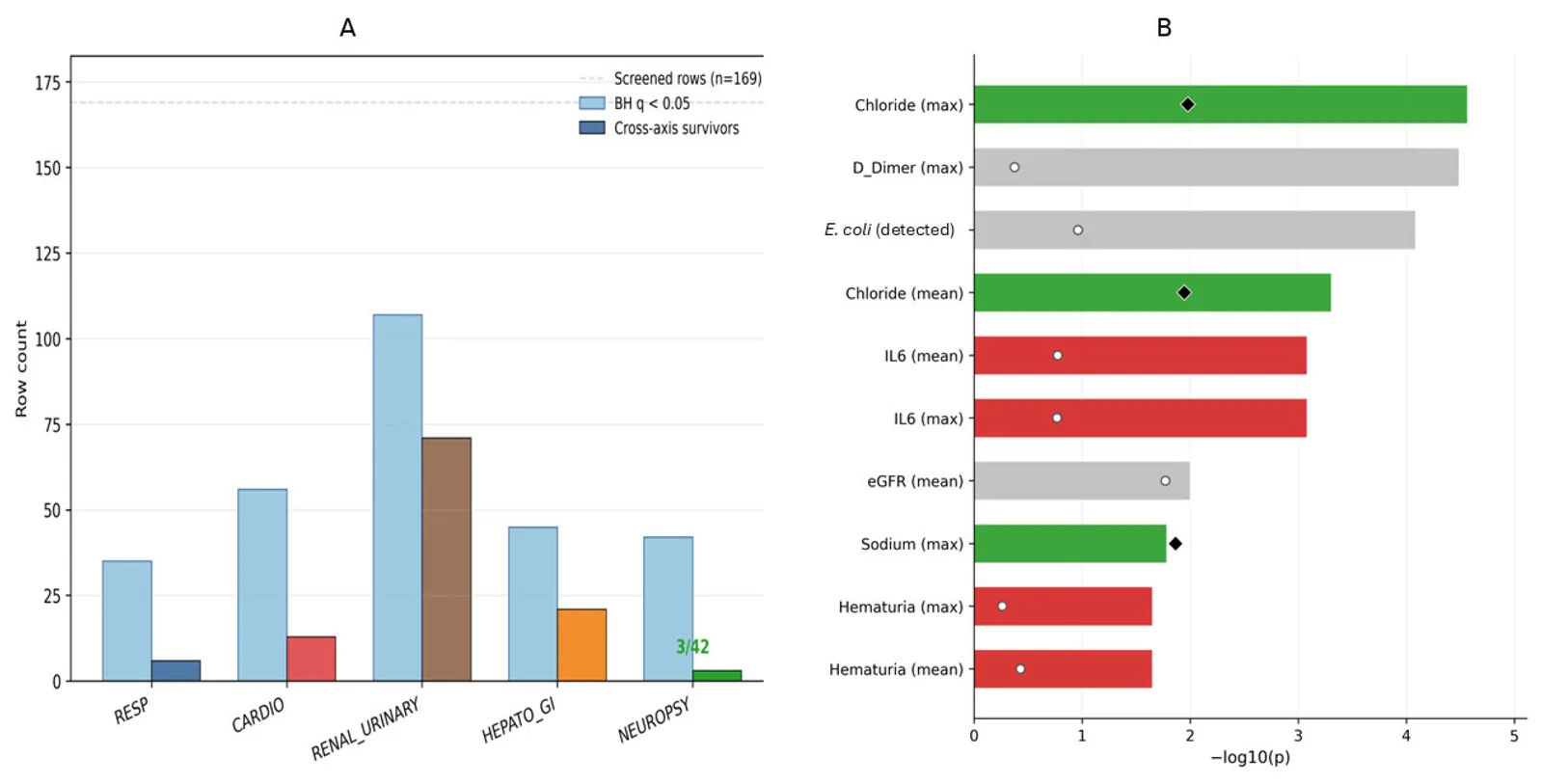
(**A**) NEUROPSY paraclinical discovery funnel: row-level bivariate screening, false-discovery-rate filtering, and cross-axis-adjusted survivors across organ axes. Fractions in panel A denote, for each axis, the number of associations retained after cross-axis adjustment over the number of candidates significant at the bivariate false-discovery-rate screen (q < 0.05); for the neuropsychiatric axis, 3/42 indicates that three of 42 such candidates were retained. In panel A, light-blue bars show the candidates significant at the bivariate screen for each axis, the adjacent bars in the axis colour show the cross-axis survivors, and the dashed line marks the 169 screened rows. (**B**) Neuropsychiatric laboratory candidates before and after cross-axis adjustment. Bars show the strength of each bivariate association (−log_10_ of the false-discovery-rate-adjusted q value). Green bars denote the three associations retained after adjustment for the other four organ axes; red bars denote the interleukin-6 and hematuria exemplars discussed in the text; grey bars denote the remaining demoted candidates. Filled diamonds mark the adjusted *p* value for retained associations, and open circles mark the adjusted *p* value for demoted candidates, illustrating the collapse of statistical support after adjustment.

Neuropsychiatric subcluster death models (NE2, NE3, PSY1, PSY2, NEPSY3) were fit separately with Firth penalization, adjusting for age, sex, and pandemic wave only (Figure S2). A post-hoc NE3 sensitivity analysis was performed using the cross-axis framework presented in Figure 3. Laboratory models included the other four organ axes, age, sex, hospitalization duration, and pandemic wave. The Late Omicron model included age and sex, with the other four organ axes added in the sensitivity specification, whereas the death and intensive care admission models included age, sex, and pandemic wave, followed by the other four organ axes. These analyses were not prespecified and are reported in Figure S3.

### 2.7. Missing Data and Statistical Software

Distributional assumptions were examined for the laboratory parameters entering bivariate testing; all testable parameters departed from normality. Non-estimable, sparse, and separation-sensitive terms were retained in output tables as explicit nulls or warnings instead of being dropped. Analyses were performed in Python 3.14 using pandas (v3.0.0), NumPy (v2.5.0), SciPy (v1.17.0), statsmodels (v0.14.6), and scikit-learn (v1.8.0), with spatial extensions where applicable. Significance was two-sided at α = 0.05 unless otherwise noted for FDR layers.

## 3. Results

The analytic cohort comprised 1361 adult patients hospitalized with confirmed COVID-19. Median age was 66 years, 54.6% were female, 14.1% required intensive care unit (ICU) admission, and in-hospital mortality was 9.4% (Figure S1; Table S4).

### 3.1. Multi-Organ Phenotype Atlas

The five-axis phenotype structure was dominated by non-neuropsychiatric organ involvement. The RESP axis was near-universal (96.8%), followed by CARDIO, HEPATO_GI, RENAL_URINARY, and NEUROPSY (Table 1). The neuropsychiatric axis was clinically common but almost never the dominant phenotype: although 312 patients were NEUROPSY-positive, only 11 (0.8%) were classified as NEUROPSY-dominant (Figure 1).

Multi-axis morbidity was frequent, with a total of 1239 patients (91.0%) across at least two organ axes. Under the conservative dominance rule 379 patients (27.8%) were classified as MULTI_MORBID dominant phenotype, whereas RESP was the most frequent single dominant label (Table 1). Among NEUROPSY-positive patients, 98.1% carried at least one additional chronic axis, and the NEUROPSY state split was predominantly chronic (236 chronic, 57 acute, 19 acute-on-chronic), arguing against the interpretation of broad NEUROPSY positivity as acute neuro-COVID biology in isolation.

Cross-axis overlap analysis showed that the highest non-respiratory pairwise co-occurrence was CARDIO with RENAL_URINARY (Jaccard 0.404 in the RESP-excluded matrix). Any-state respiratory–cardiovascular overlap was higher (Jaccard 0.745), reflecting the near-universal prevalence of the respiratory axis. Acute and acute-on-chronic neuropsychiatric overlap with other acute axes was uniformly low, whereas chronic-state overlap was substantially higher (Table S3).

### 3.2. Neuropsychiatric Hypothesis Set

Prior to multi-organ adjudication, previously defined neuropsychiatric subphenotypes [24] were evaluated for multivariate paraclinical discrimination in the laboratory-matched inpatient cohort.

In this initial discovery phase, NE3 showed the strongest discrimination. The primary NE3 model (Variant A) achieved an area under the receiver operating characteristic curve (AUC) of 0.822. A stricter NE3 definition requiring convergent documentation from at least two independent detection methods (Variant B) reached AUC 0.837, and substituting oxygen-support level for ICU admission in the covariate set (oxygen-support sensitivity; *n* = 877) retained AUC 0.813. PSY1 discrimination was moderate (Variant A AUC 0.741), whereas broad NEUROPSY positivity was weaker (AUC 0.708). NE2, the milder neurological counter-phenotype, showed only modest discrimination (AUC 0.673), consistent with a less severe paraclinical profile than NE3 (Table S1).

Across models, retained predictors were not confined to a neuropsychiatric-specific laboratory domain. NE3 models drew on coagulation, renal and metabolic injury markers, hematological indices, muscle-injury and hepatic parameters, and selected microbiological detections. PSY1 and NEUROPSY models were dominated by coagulation, inflammation, and hematological variables rather than neuropsychiatric-specific analytes. These patterns were visible at the screening stage and did not, by themselves, establish neuropsychiatric-specific biology: they indicated that apparently strong paraclinical discrimination could be reproduced across sensitivity constructions while still reflecting multi-organ systemic illness.

These models defined the hypothesis set carried forward to adjudication: reproducible discrimination across NE3 sensitivity variants, but with predictor composition spanning renal, cardiovascular, hematological, and hepatic domains, which motivated the cross-axis adjustment reported below.

### 3.3. Residual Neuropsychiatric Laboratory Signal After Cross-Axis Adjustment

NEUROPSY was screened across 169 laboratory and microbiology row-level comparisons, with 42 meeting Benjamini–Hochberg q < 0.05 at the bivariate layer (Figure 3A,B). After logistic models simultaneously adjusted for the other four organ axes plus age, sex, hospitalization duration, and pandemic wave, three associations were retained as neuropsychiatric candidates: chloride mean (adjusted OR 1.20, *p* = 0.011), chloride max (adjusted OR 1.21, *p* = 0.011), and sodium max (adjusted OR 1.19, *p* = 0.014; Table S6; Figure 3B). Mean estimated glomerular filtration rate also remained nominally significant after the same adjustment (adjusted OR 1.33, *p* = 0.017) but was adjudicated as renal-urinary-specific and is reported in Table S6 as a demotion exemplar rather than as a neuropsychiatric-specific association. Candidate signals that had appeared prominent in the hypothesis set or in bivariate screening did not survive NEUROPSY cross-axis adjustment. IL-6 illustrated this pattern most clearly. Max and mean values were q-significant for NEUROPSY in bivariate testing and were labeled NEUROPSY-specific in the screening layer, yet neither remained significant after cross-axis adjustment (adjusted *p* = 0.171 and 0.169, respectively).

The three surviving electrolyte rows were not interpretable as a narrow neuropsychiatric biomarker signature. In bivariate fingerprinting, all three were classified as multi-axis, not NEUROPSY-specific; chloride mean showed its strongest bivariate association with RESP, whereas chloride max and sodium max showed their strongest associations with RENAL_URINARY. At the axis level, NEUROPSY positivity remained independently associated with RENAL_URINARY burden after mutual adjustment (OR 1.37, 95% CI 1.04–1.82, *p* = 0.027), whereas CARDIO was not independently associated (OR 0.91, *p* = 0.59) and HEPATO_GI showed only a borderline inverse association (OR 0.77, *p* = 0.060). RESP showed a strong inverse association with NEUROPSY in the same model (OR 0.32, *p* = 0.001), consistent with NEUROPSY occurring predominantly within broader multi-organ admission profiles rather than as a respiratory-dominant phenotype.

Taken together, these results indicate that the apparent breadth of neuropsychiatric paraclinical associations in preliminary analyses largely reflected shared systemic and renal-axis burden. That the same adjustment framework retained 71 of 107 candidates for the renal-urinary axis (Figure 3A) demonstrates a marked asymmetry between axes; however, it does not exclude differential attenuation related to shared disease severity, axis prevalence, or documentation structure. The residual sodium and chloride associations are therefore reported as renal-electrolyte co-associations, not as standalone neuropsychiatric biology.

Because neuropsychiatric axis positivity was predominantly chronic, the same cross-axis covariate set was applied post hoc with the severe neurological subcluster NE3 (*n* = 132) as the outcome (Figure S3). Of 58 estimable models, three rows met the same survivor rule (hematuria mean, adjusted OR 1.49, 95% CI 1.23–1.80; hematuria max, OR 1.41, 95% CI 1.18–1.69; neutrophil percentage max, OR 1.28, 95% CI 1.02–1.61), whereas sodium max, chloride max, uric acid, INR, and IL-6 did not (Figure S3B). These residual rows were not passed through the treatment- and indication-bias gates applied to axis-level candidates (Table S7) and are not interpreted as neuropsychiatric-specific biomarkers.

### 3.4. Bias-Control Gates and Informative Null Results

For the broad NEUROPSY axis, cross-axis adjustment left only three electrolyte co-associations and demoted hematuria, IL-6, coagulation markers, uric acid, and microbiological detections that had appeared promising in bivariate screening (Figure 3; Table S6). The residual candidates were subjected to four prespecified bias-control modules: medication and indication confounding, radiology circularity control (ensuring that imaging findings used to define a phenotype could not also serve as predictors of the same phenotype), county-wide geographic testing, and pathway mediation review. This was done to determine whether associations that survived statistical adjustment still retained interpretable biological meaning once treatment exposure, imaging selection, referral geography, and temporal ordering were explicitly considered.

#### 3.4.1. Treatment and Indication Confounding

The five laboratory and microbiology candidates entering this gate were not selected because a neuropsychiatric mechanism had been proposed for any of them; they were selected by penalized regression from the full measured laboratory and microbiology space, and the purpose of this module was to establish whether any could bear a neuropsychiatric reading once treatment and testing indication were held constant. None did. Models that additionally adjusted for anticoagulant, antibiotic, corticosteroid, and severity-indication proxies showed that several associations tracked care pathways rather than NEUROPSY-specific biology. The hematuria association attenuated after adjustment for anticoagulant exposure and other treatments and was retained only as a renal-coagulation bridge variable, consistent with its stronger signal on the renal axis than on the neuropsychiatric axis (Figure 3B; Table S7).

*Klebsiella pneumoniae* and IL-6 were classified as indication-sensitive: their apparent NEUROPSY associations aligned with antibiotic prescribing and inflammatory measurement patterns tied to disease severity rather than to a discrete neuropsychiatric mechanism. Coagulation indices (INR, D-dimer, prothrombin activity) behaved as treatment and protocol-linked markers rather than independent neuropsychiatric predictors. Uric acid remained proxy-limited renal-metabolic context because medication and renal-function proxies did not support a standalone neuropsychiatric interpretation (Table S7).

Two of the five candidates did not merely lose significance but reversed direction under treatment and indication adjustment: coagulation indices moved to an adjusted OR of 0.49 (*p* = 6.2 × 10^−4^) and IL-6 to 0.31 (*p* = 0.005) (Table S7). Because both markers are measured and treated according to severity, these reversals are most consistent with treatment and indication effects and were not assigned a mechanistic interpretation (Table S7).

#### 3.4.2. Radiology Circularity Control

A dedicated radiology audit separated imaging variables used to define organ-axis phenotypes from those permitted as independent predictors of the same axis. When cranial and thoracic imaging findings that had been associated with neuropsychiatric outcomes in the initial exploratory radiology analyses were re-tested under this rule, none were retained as independent NEUROPSY predictors. For NEUROPSY specifically, brain atrophy, edema, hemorrhage, hydrocephalus, leukoaraiosis, chronic stroke, cranial burden scores, and related severity gradings were blocked because they overlapped the neuropsychiatric phenotype definition or acted as reference-only findings. Cranial CT frequency and composite neuroimaging indicators were reclassified as indication and selection proxies and not as evidence of NEUROPSY biology (Table S8).

#### 3.4.3. Geographic and Spatial Null Results

County-wide analyses accordingly tested whether organ-axis positivity clustered by road distance to hospital, altitude, urban versus rural residence, or local spatial autocorrelation. NEUROPSY showed no distance, altitude, urban/rural, or FDR-significant local-cluster association; the module therefore returned an informative null for neuropsychiatric geography in this cohort (distance OR 1.01, *p* = 0.155; no false-discovery-rate-significant local units). RESP, CARDIO, and RENAL_URINARY were also spatial nulls. HEPATO_GI was the only axis with both a distance association (*p* = 0.002) and two significant local units; this was not investigated further and may reflect catchment, referral, or local exposure patterns (Table S9).

#### 3.4.4. Pathway Mediation Review

Prespecified exposure–mediator–outcome paths were tested to avoid upgrading associative chains to causal mediation language (Table S10). The hospitalization duration → ICU admission → NE3 symptom-cluster path was estimable only as exploratory attenuation (13% attenuation of the exposure–outcome association after introducing ICU) and was not promoted as mediation because both hospitalization duration and ICU admission are post-admission care constructs with indefensible temporal ordering for causal inference. Two uric-acid-centered mediation paths (pandemic wave → uric acid → NE3; ICU → uric acid → NE3) were not estimable in the integrated model frame and were preserved as informative nulls. No prespecified pathway met the criteria for retention as a mediation claim.

Collectively, these bias-control modules converged on the same conclusion as the cross-axis laboratory screen. Apparent NEUROPSY paraclinical and symptom-based radiology signals largely reflected shared systemic illness, treatment exposure, imaging selection, and renal-axis burden. Previously highlighted positive associations were therefore downgraded when stricter, clinically grounded controls were applied (Tables S5 and S7–S10).

### 3.5. ICU Admission and Mortality Outcome Hierarchy

Renal-urinary and cardiovascular axis positivity carried the strongest independent associations with ICU admission and in-hospital death after mutual adjustment for all five organ axes, whereas broad neuropsychiatric axis positivity did not retain a significant adjusted association with either endpoint (Table 2; Figure 2; Table S11). Outcome models used binary axis-positivity flags assigned from ICD evidence, not the mutually exclusive dominant-phenotype labels. Renal-urinary axis positivity (has_RENAL_URINARY = 1) applied to 513 admissions with 87 deaths, whereas only 23 admissions were renal-urinary-dominant and none of those died as the assigned dominant phenotype.

Across the five axis flags, renal-urinary positivity was the only term with stable significance for both ICU admission and death (Table 2; Table S11). Cardiovascular axis positivity (has_CARDIO = 1) was present in 127 of 128 decedents; the adjusted death association was strong under penalized estimation (Firth OR 19.4) but did not translate into a significant ICU association (OR 1.43, *p* = 0.116). Respiratory axis positivity was near-universal (96.8%); the 44 admissions without coded respiratory-axis ICD evidence (has_RESP = 0, 3.2%) included no deaths, so the maximum-likelihood death odds ratio was not identifiable; the penalized estimate (Firth OR 6.49, 95% CI 0.86–832) spans unity and is reported in Table S11 rather than as a quantitative effect. Hepato-GI positivity was non-significant for both endpoints. Broad neuropsychiatric axis positivity showed a borderline ICU association in the primary model (OR 1.39, *p* = 0.072) that was non-significant after hospitalization-duration adjustment (OR 1.23, *p* = 0.270); the adjusted death OR was 1.33 (*p* = 0.20), compared with an unadjusted OR of 1.75 (*p* = 0.008).

In separate models for neuropsychiatric subclusters, no subcluster retained a significant independent death association after penalized estimation (Figure S2). NE3 (Firth OR 1.70, *p* = 0.060) and PSY2 (Firth OR 2.15, *p* = 0.065; *n* = 43) were borderline, the latter within the range flagged as underpowered; NE2 and PSY1 were null; NEPSY3 (*n* = 12) remained underpowered. In a post-hoc extension of the NE3 death model that additionally adjusted for the other four organ axes, the Firth odds ratio moved from 1.70 (95% CI 0.98–2.87, *p* = 0.060) to 1.60 (95% CI 0.90–2.74, *p* = 0.105) (Figure S3C). The corresponding models for intensive care admission gave 2.63 (95% CI 1.69–4.04) before and 2.51 (95% CI 1.61–3.89) after four-axis adjustment (Figure S3C).

### 3.6. Era Trajectories and the Late Omicron/NE3 Robustness Check

**Figure 4:**
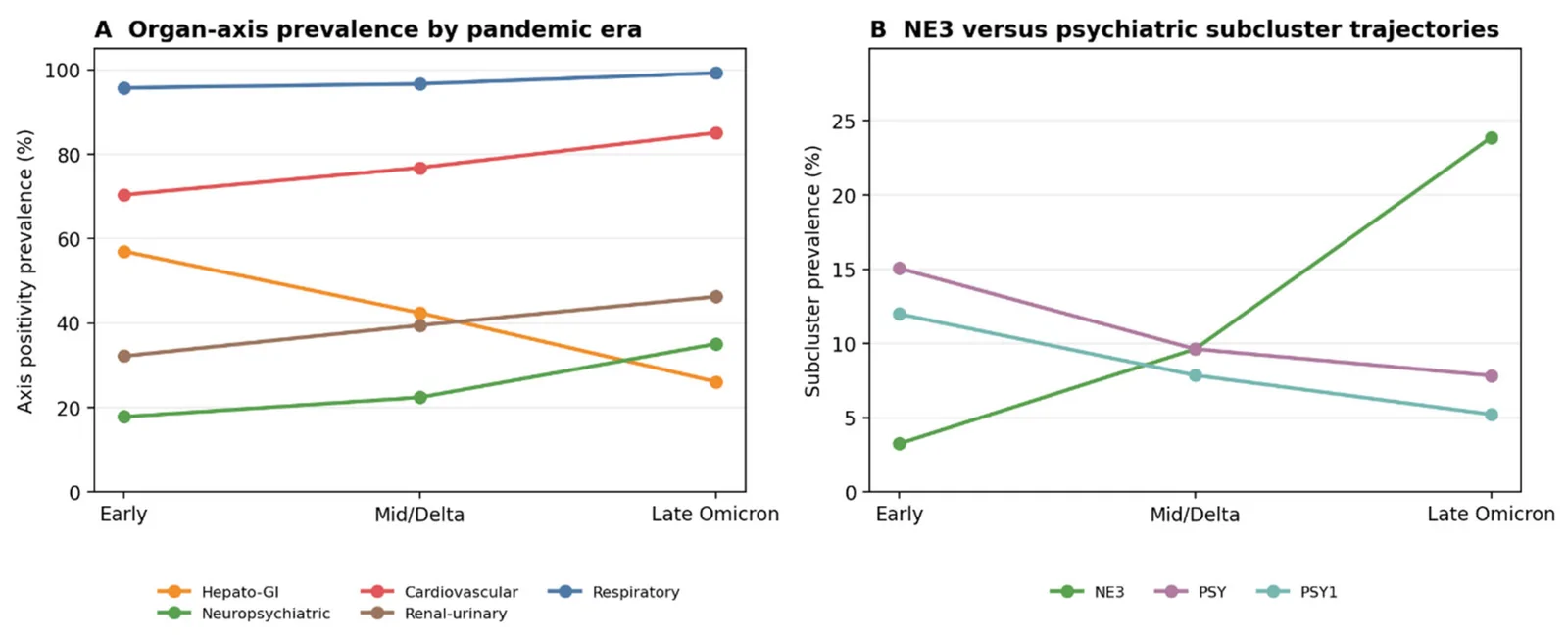
Pandemic-era trajectories of organ-axis and neuropsychiatric subcluster prevalence. (**A**) Prevalence of the five organ axes across the Early, Mid/Delta, and Late Omicron periods. (**B**) Prevalence of neuropsychiatric subclusters across the same periods, showing an increase in NE3 alongside declining PSY and PSY1 prevalence. Adjusted Late Omicron odds ratios are reported in Table S2.

Organ-axis and neuropsychiatric subcluster prevalence shifted unevenly across pandemic eras (Figure 4). Hepato-gastrointestinal axis positivity fell from Early through Late Omicron admission periods, whereas neuropsychiatric axis positivity rose (17.8% to 35.1%; adjusted OR 1.57, *p* = 0.004); cardiovascular, renal-urinary, and respiratory axes showed no clear adjusted Late Omicron shift after age and sex adjustment (Figure 4A; Table S2). Era-stratified testing compared all five organ axes before interpreting neuropsychiatric temporal patterns.

At subcluster level, the Late Omicron increase was concentrated in NE3 and not distributed across all neuropsychiatric labels: NE2, broad NEURO, PSY2, and NEPSY3 showed no clear positive era shift, while PSY and PSY1 declined over the same periods (Figure 4B; Table S2). This pattern contrasts the rising NE3 trajectory with falling psychiatric-era prevalence rather than treating neuropsychiatric labels as a single temporal block. The Late Omicron odds ratio for NE3 in Table S2 (3.35) is adjusted for age and sex. In a post-hoc model that additionally adjusted for the other four organ axes, the estimate was essentially unchanged (OR 3.36, 95% CI 2.25–5.02; Figure S3C).

### 3.7. Final Claim Re-Tiering and Synthesis

Thirty-five prespecified claims from the initial hypothesis set were adjudicated through the integrated analysis pipeline. At final integration, 22 claims were refuted or not retained, five were relabeled as shared multi-axis markers, three were treatment or protocol-linked, two were spatial context only, two were underpowered, and one was retained as neuropsychiatric-specific (Table S12).

The adjudicated evidence describes a single-center hospitalized COVID-19 multi-organ phenotype atlas: neuropsychiatric axis positivity was common but rarely dominant; broad neuropsychiatric paraclinical associations narrowed to a renal-electrolyte co-association after mutual organ-axis adjustment; bias-control modules demoted treatment-, radiology-, spatial-, and mediation-linked candidates; adjusted intensive care and mortality burden fell mainly on renal-urinary and cardiovascular axis positivity; and the previously reported Late Omicron enrichment of severe neurological presentations persisted after age and sex adjustment and, post hoc, after adjustment for four concurrent organ axes, as the single claim retained as neuropsychiatric-specific (Table S12).

## 4. Discussion

In this retrospective hospitalized COVID-19 cohort, record-derived neuropsychiatric involvement was common but almost never the dominant phenotype, and nearly all affected patients carried concurrent chronic disease in at least one other organ system. Under prespecified adjudication, neuropsychiatric paraclinical and prognostic associations did not persist as organ-specific once concurrent involvement, treatment exposure, and documentation structure were held constant, whereas renal-urinary involvement retained independent associations with both outcomes.

Three features of this pattern structure the interpretation that follows: neuropsychiatric labels are nested within multimorbid admissions rather than defining standalone phenotypes; the laboratory associations that survived adjustment describe a shared renal-metabolic axis rather than a neuropsychiatric biomarker set; and the adjusted prognostic hierarchy places renal-urinary involvement ahead of neuropsychiatric involvement. The contribution is therefore one of reinterpretation and framework rather than biomarker discovery.

Under fixed-order testing, most bivariate neuropsychiatric laboratory, microbiology, radiology, spatial, and mediation-linked patterns were more consistent with shared systemic severity, treatment exposure, and documentation structure than with neuropsychiatric-specific effects. Of 35 prespecified claims, one retained neuropsychiatric-specific status. The remainder were demoted, relabeled as shared context, or flagged as underpowered. This attrition delineates the boundary between association and neuropsychiatric-specific inference.

### 4.1. Neuropsychiatric Phenotypes in a Multi-Organ Atlas

Early hospitalized cohorts showed that neurological manifestations frequently accompanied systemic COVID-19 illness rather than occurring in isolation: 36.4% of inpatients in the first Wuhan series had neurological manifestations, more often in severe disease [6], and a later prospective series reported presentations in 23.4%, with renal and inflammatory laboratory divergence by presentation type but no severity gradient specific to neurological symptoms [7]. Such symptom-defined cohorts support the clinical relevance of neuropsychiatric presentations but do not map directly onto record-derived organ axes. Consistent with external work treating COVID-19 laboratory panels as broad systemic prognostic tools [1,29], the initial multivariate models here retained no analyte with established neurological specificity, and biomarker studies linking neuroinflammatory markers to severity and neuroimaging findings drew on complication-enriched populations that are not directly comparable with an unselected hospitalized cohort [25].

The acuity-stratified overlap structure locates the embedding: neuropsychiatric co-occurrence with other axes was markedly lower in the acute matrix and substantially higher in the chronic matrix (Table S3), indicating a chronic comorbidity scaffold rather than simultaneous acute multi-organ failure. The chronic component of the neuropsychiatric axis therefore primarily captures documented pre-existing disease rather than acute neuro-COVID. External evidence supports separating that chronic burden from acute severity: in a propensity-matched Brazilian cohort from 38 hospitals, pre-existing dementia did not account for short-term mortality after severe COVID-19 [30].

Prognostic literature on chronic neuropsychiatric burden points the same way. An umbrella review found increased COVID-19 mortality in people with pre-existing mood and schizophrenia spectrum disorders without clear evidence of increased severity, attributing the excess to cardiovascular comorbidity, psychotropic exposure, and restricted access to high-intensity care [31]. In 6036 hospitalized patients phenotyped from ICD-10 codes, dementia carried an adjusted in-hospital mortality odds ratio of 1.44 that rose with the number of coexisting conditions [32], whereas in a multicenter cohort with age modeled flexibly, only diabetes, chronic kidney disease, and chronic obstructive pulmonary disease remained independently associated with death [33]. The present pattern, of an unadjusted neuropsychiatric mortality association that did not persist after mutual adjustment, alongside a renal signal that did, is consistent with this literature.

### 4.2. Paraclinical, Microbiological, and Imaging Associations After Cross-Axis Adjustment

Cross-axis adjudication demoted most laboratory, microbiology, and imaging associations identified at the bivariate stage. Inflammatory, coagulation, and neural-injury markers (IL-6, LDH, ferritin, and troponin) are repeatedly elevated in severe COVID-19 with neurological presentation and have been linked with mortality in neurological inpatients [12]. CRP, ferritin, and D-dimer show a similar pattern in stroke cohorts [34], with fibrinogen, D-dimer, and CRP implicated along inflammation–coagulation pathways in Omicron-wave stroke [35], and GFAP/NfL in prognostic meta-analysis [13]. In the prespecified broad-NEUROPSY analysis, IL-6, coagulation parameters, hematuria, uric acid, and renal proxies lost NEUROPSY specificity after mutual organ-axis adjustment and treatment or indication review. They are therefore more consistent with systemic severity markers than with compartment-specific biomarkers.

Demotion should not be read as clinical irrelevance. Renal abnormalities and coagulation markers are established correlates of COVID-19 severity and prognosis [36,37,38,39], but their associations did not retain neuropsychiatric specificity after concurrent organ involvement and treatment or indication effects were considered. Because candidates were selected statistically rather than on the basis of a prespecified neuropsychiatric mechanism, demotion concerns specificity rather than mechanistic relevance. Residual confounding remains possible, particularly for the nominally retained electrolyte associations.

Microbiology and imaging followed the same logic. Bacterial detections in COVID-19 frequently mark nosocomial exposure, antimicrobial pressure, and severity pathways [40,41]. Neuroimaging reports substantial abnormality rates among selected patients [25,42] but high between-study heterogeneity [26,43]. Denominator structure is decisive: in an 8675-patient cohort, only 2.07% underwent head imaging and 86% of imaged examinations were normal [44]. Radiological findings here were more consistent with work-up intensity and indication bias than with an independent NEUROPSY-specific association, consistent with early CT/MRI series emphasizing secondary immune and vascular mechanisms [45].

The three nominally retained electrolyte associations (mean chloride, maximum chloride, and maximum sodium) are best interpreted collectively as a renal-metabolic/electrolyte co-association rather than as independent neuropsychiatric biomarkers. Neuron-specific enolase and electroencephalographic patterns likewise stratify encephalopathy severity in severe pneumonia without establishing neuropsychiatric specificity once multi-organ disease is controlled [19]. Ascertainment context adds a separate layer: recorded delirium prevalence in geriatric COVID-19 hospitalizations shifted substantially with the introduction of dexamethasone-era treatment protocols [46], showing that care-process change alone can alter measured neuropsychiatric prevalence. Reproducible bivariate record-derived associations can therefore fail neuro-specific adjudication once multi-organ co-occurrence, indication, and care-process structure are explicitly considered.

### 4.3. Renal-Urinary and Cardiovascular Prognostic Burden

Where paraclinical neuro-specific claims were demoted, mutually adjusted outcome models identified a different prognostic hierarchy. Dominant-phenotype tables and axis-positive outcome models answer different questions: a patient can carry renal-urinary ICD evidence while the dominant phenotype is respiratory, cardiovascular, or multimorbid. Outcome models therefore estimate the adjusted association of axis positivity within multimorbid admissions rather than outcomes in the renal-dominant subgroup alone.

On that estimand, renal-urinary positivity was the only axis with stable significance for both ICU admission and death, with close agreement between maximum-likelihood, Firth, and MAP estimates. The direction is externally plausible: kidney involvement in severe COVID-19 proceeds through interacting inflammatory, endothelial, microvascular, and hemodynamic mechanisms, with direct viral tropism for renal cells still contested [47], and none of these pathways were tested here. Acute kidney injury developed in 39% of a London inpatient cohort, with adjusted 30-day mortality rising across stages [48], and in 36.6% of admissions in a large US cohort [49]; these AKI-defined cohorts support directional plausibility rather than construct equivalence, because the renal-urinary axis derives from ICD documentation rather than KDIGO criteria. Lung–kidney and heart–kidney crosstalk [39] and post-mortem multi-organ endotheliitis [50] provide shared substrates for the multi-organ pattern.

Cardiovascular positivity was present in nearly all decedents: only one cardiovascular-negative admission resulted in death. Consequently, the reference cell contained a single event, and although Firth penalization reduces sparse-data bias, it cannot restore information that the data do not contain [51]. The resulting estimate should therefore be interpreted as a strong but separation-sensitive association rather than as a precise effect size. Cardiovascular positivity was not significantly associated with ICU admission.

This hierarchy is compatible with literature placing neurological involvement within broader systemic COVID-19 severity [14] and with reviews framing acute brain dysfunction within organ failure, inflammation, and blood–brain barrier disruption [8,9]. The unadjusted neuropsychiatric mortality association did not persist after mutual adjustment, resulting in an adjusted null rather than clinical irrelevance.

The cross-axis models estimate each association conditional on the other documented organ axes, modeled simultaneously as separate covariates, but they cannot determine whether those axes represent separate confounding domains or correlated manifestations of a common upstream process such as overall illness severity. In the latter situation, simultaneous adjustment may constitute overadjustment by conditioning on correlated manifestations of the same severity process, thereby mechanically attenuating a genuine neuropsychiatric-specific signal rather than revealing its absence. The sharp asymmetry between the neuropsychiatric (3 of 42) and renal-urinary (71 of 107) associations retained under the same cross-axis adjustment framework is consistent with either reading: neuropsychiatric positivity may carry less organ-specific biology, or its signal may be absorbed more completely by a shared severity pathway that the renal axis indexes more directly. The present data cannot separate these possibilities. The adjusted nulls reported here therefore show that neuropsychiatric associations do not persist conditional on concurrent organ involvement; they do not establish that such associations are absent.

Because neuropsychiatric axis states were predominantly chronic, broad positivity chiefly reflects pre-existing disease, and the 76 acute or acute-on-chronic axis-positive admissions were too few to support a separate analysis based on axis acuity. The symptom-defined NE3 cluster (*n* = 132) represents a different construct: 42 of 132 NE3 admissions were NEUROPSY-axis negative, and only 43 overlapped ICD acute or acute-on-chronic evidence. A post-hoc NE3 analysis under the same cross-axis rule is reported in Figure S3; nevertheless, the broad-axis analysis may still dilute a distinct acute or severe neurological signal.

### 4.4. Pandemic-Era Shifts and the Multi-Organ Sensitivity Analysis

The temporal contrast is treated here as a robustness check rather than as a new temporal discovery. NE3 increased from 3.3% in the Early period to 23.9% in the Late Omicron period (age- and sex-adjusted OR 3.35), while PSY1 and HEPATO_GI declined, reproducing within an organ-axis framework the wave-level divergence previously reported for this cohort [24]. Adding the other four organ axes left the NE3 estimate essentially unchanged (OR 3.36, 95% CI 2.25–5.02; Figure S3C); this conditional stability, rather than the divergence itself, is the contribution of the present analysis.

Polish wave-stratified cohorts documented shifting neurological spectra from pre-Delta through Omicron, including later rises in delirium, transient ischemic attack, and encephalopathy despite lower overall COVID-19 severity [10,11]. An Omicron-wave neuroimmune case series [52] and multicenter studies of COVID-associated neurological syndromes from earlier pandemic periods [53], together with variant-period severity and vaccination data [54], reinforce that era comparisons mix viral biology, population immunity, and care context. Long-COVID neurological reviews further support caution when transporting imaging or biomarker findings across acute and post-acute settings [55]. External literature therefore provides plausibility context rather than validation of the NE3 construct.

The era contrast also carries a specific potential confound. From the Omicron period onward, hospital SARS-CoV-2 positivity increasingly included patients in whom COVID-19 was contributing or incidental rather than the primary reason for admission; this was documented for BA.1/BA.2 admissions [56] and there is no reason to expect it to have reversed under the later sublineages that dominate the Late Omicron period here. Part of the Late Omicron NE3 enrichment may therefore reflect a shift in admission indication, with patients hospitalized primarily for neurological or psychiatric conditions testing positive on screening. Adjustment for concurrent organ axes, including the four-axis models in Figure S3C, does not identify why a patient was admitted, and the record-derived data used here cannot distinguish reliably between admission with and admission for COVID-19, so this explanation cannot be excluded. Late Omicron/NE3 should therefore be reported as an internally adjudicated temporal enrichment worth replication, not as evidence of variant neurotropism or elevated NE3 mortality risk.

### 4.5. Prespecified Claim Adjudication and Informative Nulls

Prespecified fixed-order adjudication, rather than post-hoc filtering, determined which initial associations were retained, demoted, or classified as underpowered (Table S12). Across the 35 prespecified claims, the adjudicated evidence did not support neuropsychiatric-specific biomarker associations, an independent adjusted mortality association, mediation-based mechanistic inference, radiology-based mechanisms, or axis-level spatial clustering; one claim retained neuropsychiatric-specific status. Negative radiology, spatial, mediation, and treatment-linked modules are therefore informative because they identify where initial record-derived associations did not meet the prespecified criteria for neuropsychiatric-specific inference.

Such attrition does not imply absence of clinically relevant neuropsychiatric burden. Longer-term follow-up after COVID-19 hospitalization has demonstrated persistent and evolving cognitive and psychiatric morbidity, with depression, anxiety, and fatigue worsening or newly emerging in some patients at 2–3 years [57]. Conversely, in an acute-care multicenter comparison of SARS-CoV-2-positive and -negative respiratory infections, delirium incidence was comparable and no substantial COVID-19-specific neurocognitive impact was identified across ages and disease severity [58]. Together, these apparently contrasting findings illustrate how neuropsychiatric associations depend on population, clinical context, and time horizon rather than uniformly indicating COVID-19-specific neurological biology.

In the present record-derived analysis, systemic illness, testing behavior, and documentation context remain plausible explanations for many initial associations [4,5]. Prespecified adjudication makes explicit where those associations persist and where they do not support a more specific neuropsychiatric interpretation.

### 4.6. External Evidence and Analytic Comparability

Most external neuro-COVID studies pose a different question. Neurologist-referral registries such as ENERGY document complication spectra among referred inpatients [20,21]; symptom-ascertained cohorts estimate manifestation rates among patients assessed neurologically; and cerebrospinal fluid, proteomic, and imaging-enriched samples examine biology in complication-selected subsets. Longitudinal hospitalized cohorts [57], population meta-analyses [3], and long-COVID neurological reviews [55] address post-discharge burden, while large electronic health record frameworks show that post-acute phenotype prevalence depends on coding windows and surveillance structure as well as underlying biology [4,5]. Each of these designs serves its own purpose better than the present one could: characterizing neurological spectra, quantifying symptom burden, and probing mechanism all require ascertainment depth that whole-admission record-derived phenotyping cannot supply. Conversely, none directly estimates the contrast examined here, in which axis positivity is evaluated with co-occurring organ involvement held constant (Table S13). Directional concordance therefore supports clinical plausibility without corroborating this hospital’s effect sizes, and discordance more likely reflects phenotype definition and indication selection than genuine inconsistency between studies.

Within those limits, two implications follow. Clinically, documented neuropsychiatric involvement in hospitalized COVID-19 identifies patients with substantial chronic multi-organ comorbidity rather than a group at independently elevated risk of death; concurrent renal-urinary involvement carries the adjusted prognostic weight, and an electrolyte and renal-function assessment may be a more defensible response to such documentation than a search for neuropsychiatric-specific biomarkers. The prespecified sequence used here—cross-axis adjustment → treatment and indication gates → outcome models, with auditable claim attrition—offers a transferable template for any record-derived phenotype in which co-occurring organ disease is the rule rather than the exception.

### 4.7. Limitations

This single-center, retrospective, record-derived study is subject to missing or incomplete data, misclassification, indication-driven measurement, unmeasured confounding, and limited generalizability beyond hospitalized patients with COVID-19. Because exposures and phenotypes were reconstructed from routine hospital records rather than protocol-driven assessments, their accuracy and completeness depend on how thoroughly clinicians documented findings at the time of care.

Four limitations bear directly on the interpretation above. First, the organ axes were constructed from ICD-linked diagnoses, extracted text, and clinical documentation rather than biological compartments, and were not validated against manual chart adjudication; reported limits to the sensitivity of ICD-10 delirium coding illustrate the potential for under-ascertainment without establishing the direction or magnitude of the resulting bias [59,60]. Second, false-discovery-rate control was applied at the bivariate screen but not repeated at the adjusted stage, so the residual electrolyte associations should be regarded as exploratory, and unavailable covariates—fever, volume status, glycemia, and coded diabetes—leave residual confounding possible for precisely those associations. Third, the cross-axis models estimate associations conditional on the other documented organ axes. If these axes partly reflect a shared severity process, simultaneous adjustment may constitute overadjustment and potentially attenuate a genuine axis-related signal. Figure S3 therefore reports minimally adjusted and four-axis estimates for the NE3 subcluster side by side, without designating either as the true effect. That post-hoc sensitivity analysis rests on 21 NE3 deaths, so a modest death association is compatible with both a null and a small positive effect; its intensive care association is a sensitivity finding rather than a primary claim; its residual laboratory rows were not treatment-gated; and it uses a symptom-defined cluster that only partly overlaps the ICD-defined acute axis states. The adjusted null findings for broad NEUROPSY positivity should therefore not be extrapolated to acute or severe neurological presentations. Fourth, linkage loss was concentrated in the later pandemic waves, and the available records could not reliably distinguish admission for COVID-19 from incidental SARS-CoV-2 positivity. Era comparisons may therefore reflect selection, admission indication, documentation practices, population immunity, vaccination, and treatment protocols alongside variant biology. Informed consent for clinical data use was obtained at hospital admission, and no additional study-specific consent was required for the anonymized retrospective analysis conducted under ethics approval. Remaining limitations, including the absence of baseline psychiatric history, discharge-only medication timing, and the shared-data nature of the penalized estimators, are itemized in Table S14.

## 5. Conclusions

In this retrospective single-center cohort of 1361 adults hospitalized with COVID-19, neuropsychiatric involvement documented in hospital records was common but nested within chronic multi-organ disease. Prespecified adjudication left no analyte adjudicated as neuropsychiatric-specific, and the adjusted prognostic weight rested on renal-urinary rather than neuropsychiatric involvement, while severe neurological and mild psychiatric presentations followed divergent trajectories across pandemic periods. Record-derived neuropsychiatric phenotypes are therefore best read within a multi-organ framework rather than as an isolated neurological syndrome. Because the phenotypes were derived retrospectively from routine hospital records, incomplete or variable clinical documentation may have affected phenotype ascertainment, thereby limiting the generalizability of the findings. Prospective multicenter studies combining standardized neurological assessment with prespecified multi-organ adjustment would establish how much of the neuropsychiatric signal observed in hospital records reflects organ-specific biology and how much reflects the systemic and documentation context in which it is recorded.

## Figures and Tables

**Table 1:** Demographic and clinical characteristics by multi-organ axis and dominant phenotype assignment.

Axis	Axis-Positive (% of Cohort)	Dominant Phenotype (%)	Age, Median		Male (%)		Stay Days, Median	ICD Codes, Median	ICU (%)		Death (%)
			Dom	Pos	Dom	Pos			Dom	Pos	
ALL	100	—	66	—	45.4	—	11	15	14.1	—	9.4
RESP	96.8	47.3	65	66	46.1	45.8	11	15	15.2	14.4	9.7
CARDIO	75.7	12.6	72	69	46.8	45.4	11	16	12.9	15.7	12.3
NEUROPSY	22.9	0.8	62	72	45.5	42.3	12	17	18.2	18.9	13.5
HEPATO & GI	45.5	9.3	55	63	46.5	42.3	12	17	5.5	15.2	10.3
RENAL & URINARY	37.7	1.7	77	71	52.2	51.8	12	18	8.7	20.9	17
MULTI MORBID *	91.0	27.8	68 *	—	43.3	—	11	—	15.8	—	12.4
NONE *	—	0.4	43	—	16.7	—	7.5	—	16.7	—	0

Values are shown as dominant-phenotype subgroup (Dom)/all axis-positive admissions (Pos) for that row where the two differ; single values apply to both. Sex is reported as percentage male (the cohort was 54.6% female). * MULTI_MORBID and NONE are dominance-only categories: the 91.0% shown for MULTI_MORBID is the proportion of the cohort positive for at least two axes, not a single-axis prevalence, and neither category has a separate axis-positive column. Axis positivity is derived from ICD-linked evidence and is not mutually exclusive; percentages therefore sum to more than 100.

**Table 2:** Mutually adjusted organ-axis associations with ICU admission and in-hospital death.

Axis	ICU Admission		In-Hospital Death	
	OR (95% CI)	*p*	OR (95% CI)	*p*
Renal_Urinary	1.97 (1.41–2.74)	5.96 × 10^−5^	2.58 (1.72–3.93) *	3.58 × 10^−6^
Cardiovascular	1.43 (0.92–2.23)	0.116	19.4 (5.2–172) *	3.39 × 10^−9^
Neuropsychiatric	1.39 (0.97–1.98)	0.072	1.33 (0.86–2.01) *	0.195
Hepato-GI	1.29 (0.93–1.78)	0.126	1.33 (0.90–1.98) *	0.153
Respiratory	1.79 (0.54–5.98)	0.345	- **	-

* Firth penalized logistic regression (profile-likelihood 95% CI, penalized LRT *p*). ** No deaths among admissions without coded respiratory-axis ICD evidence (has_RESP = 0); maximum-likelihood death OR non-identified.

## Data Availability

The data presented in this study are available from the corresponding author upon reasonable request. The data are not publicly available due to patient privacy and institutional regulations.

## Supplementary Materials

The following supporting information can be downloaded at: https://www.mdpi.com/article/10.3390/pathogens15090979/s1, Table M1: Adjudication modules; Table S1: Initial multivariate model summary; Table S2: Era prevalence and adjusted odds ratios; Table S3: Cross-axis Jaccard overlap matrices; Table S4: Cohort demographics and cluster prevalence linked versus full sampling frame; Table S5: Bias-control gate summary; Table S6: NEUROPSY cross-axis adjudication; Table S7: Treatment and indication verdicts; Table S8: Radiology reconciliation summary and NEUROPSY imaging audit; Table S9: Axis spatial verdicts; Table S10: Mediation pathway review; Table S11: ICU and death axis coefficients; Table S12: Claim adjudication summary; Table S13: Estimand literature mapping; Table S14: Itemized limitations; Figure S1: Cohort derivation with per-wave attrition and linkage representativeness; Figure S2: Neuropsychiatric subcluster death models; Figure S3: Post-hoc cross-axis sensitivity analysis for the severe neurological subcluster NE3.

## Abbreviations

The following abbreviations are used in this manuscript:

AKI	Acute kidney injury
AUC	Area under the receiver operating characteristic curve
CARDIO	Cardiovascular organ axis
CI	Confidence interval
COVID-19	Coronavirus disease 2019
CRP	C-reactive protein
CT	Computed tomography
FDR	False discovery rate
GDPR	General Data Protection Regulation
GFAP	Glial fibrillary acidic protein
HEPATO_GI	Hepato-gastrointestinal organ axis
ICD-10	International Classification of Diseases, 10th Revision
ICU	Intensive care unit
IL-6	Interleukin-6
INR	International normalized ratio
KDIGO	Kidney Disease: Improving Global Outcomes
LASSO	Least absolute shrinkage and selection operator
LDH	Lactate dehydrogenase
LRT	Likelihood-ratio test
MAP	Maximum a posteriori
MRI	Magnetic resonance imaging
MULTI_MORBID	Multi-morbid dominant phenotype
NE2	Moderate neurological subcluster
NE3	Severe neurological subcluster
NECA	Neuro-cardiovascular subcluster
NEPSY3	Combined neuropsychiatric subcluster
NEURO	Aggregate neurological indicator
NEUROPSY	Neuropsychiatric organ axis
NfL	Neurofilament light chain
OR	Odds ratio
PSY	Aggregate psychiatric indicator
PSY1	Mild psychiatric subcluster
PSY2	Moderate psychiatric subcluster
RENAL_URINARY	Renal-urinary organ axis
RESP	Respiratory organ axis
SARS-CoV-2	Severe acute respiratory syndrome coronavirus 2
STROBE	Strengthening the Reporting of Observational Studies in Epidemiology
